# “This Is Public Health: Recycling Counts!” Description of a Pilot Health Communications Campaign

**DOI:** 10.3390/ijerph6122980

**Published:** 2009-11-30

**Authors:** Nancy L.Chase, Gregory M. Dominick, Amy Trepal, Leanne S. Bailey, Daniela B. Friedman

**Affiliations:** 1 Department of Exercise Science, University of South Carolina, 921 Assembly Street, Columbia, South Carolina, 29208, USA; E-Mails: CHASEN@mailbox.sc.edu (N.L.C.); ajtrepa@mailbox.sc.edu (A.T.); 2 Department of Health Promotion, Education, and Behavior, University of South Carolina, 800 Sumter Street, Columbia, South Carolina, 29208, USA; E-Mail: gdominick75@gmail.com; 3 South Carolina Department of Health and Environmental Control, 2600 Bull Street, Columbia, South Carolina, 29201, USA; E-Mail: baileyls@dhec.sc.gov

**Keywords:** recycling, environment, health communication, public health

## Abstract

This paper describes the development, implementation, and evaluation of a pilot recycling campaign. The goal of the campaign was to increase people’s awareness and knowledge about recycling and the link between a healthy environment and the public’s health. A total of 258 individuals attended campaign week events and completed an initial survey. Results identified inconvenience of recycling facility locations as a key barrier to recycling. Post-campaign survey results revealed increased recycling of paper, plastic, glass, and cans (p < 0.05). The majority of participants “agreed” or “strongly agreed” that as a result of campaign messages they had greater awareness about recycling (88.4%) and their recycling efforts increased (61.6%).

## 1. Introduction

### 1.1. Solid Waste Concerns in the United States

The United States (U.S.) is the number one trash-producing country in the world, creating 1,609 pounds per capita on an annual average [1]. Each day in the U.S., the average person generates more than four pounds of municipal solid waste. The majority of consumers report being opposed to pollution, but rarely identify with the fact that they themselves are significant contributors to it [2]. Although it is believed that 90% of this waste is recyclable, it is estimated that less than one third of it is currently recycled, with paper comprising a large proportion of municipal solid waste [3]. In addition, Americans use 2.5 million plastic bottles every hour, most of which are thrown away [1]. Treatment of solid wastes includes landfills, incineration with heat recovery, and composting [2]. According to the U.S. Environmental Protection Agency [4], 32.1% of solid waste is recycled, 13.6% is incinerated, and 54.3% ends up in landfills. The majority of damage done to the environment and peripheral ecosystems is directly related to human behavior. With a soaring population and overproduction of material goods, this solid waste will negatively impact our surrounding environment.

### 1.2. Solid Waste Concerns in South Carolina

In 2008 in South Carolina, almost 13 million tons of solid wastes were created, and just under five million tons of solid wastes (38.5%) were recycled [5]. This number has decreased from a little over 8.5 million tons (50.2%) of recycled solid waste in 2007 [5]. Over 6.1 million tons of solid wastes were placed in landfills; 212,118 tons were placed in incinerators. The South Carolina state government developed yearly and long-term recycling goals. Unfortunately, the amount recycled in 2008 was only 24.0% of the set goal for that year [5].

By slightly modifying their actions, individuals can play a pivotal role in reducing the amount of garbage collected in their communities. Taking simple steps such as recycling plastic bottles and reusing empty cans and jars, shopping bags, and containers will help minimize unnecessary waste. According to the Department of Environmental Quality’s 2004 report, “throughout the United States, recycling has resulted in economic growth, income growth, net job increases, long-term investment, energy savings, waste reduction, lower production costs for many industries, and an extension of the life of landfills”. Furthermore, “only through a fundamental rethinking of our approach to waste can we preserve precious open space and protect our air, land, and water” [6].

### 1.3. Previous Research on Community and University Recycling

Some research has been conducted to examine if increased recycling knowledge influences individuals’ and communities’ recycling behaviors. Community-based research found that residents’ awareness of each other’s recycling habits positively affected their recycling intentions [7]. Chan also found that social norms had an effect on recycling behaviors in a public housing complex [8]. In addition to friends, relatives, and neighbors, media outlets such as newspapers, television, and magazines, had an impact on community members’ recycling behaviors. A survey of Florida residents found that individuals living in areas with greater media exposure promoting recycling reported higher levels of recycling [9].

Studies on college campuses found that increased access to recycling bins coupled with raffles and contests led to improved recycling habits among students [10,11]. Another study that provided students with recycling bins for their rooms showed a reduction in the students’ waste stream and an increase in the percentage of waste that was recycled [12]. Other studies have assessed students’ attitudes toward recycling on campus. An older study by Williams [13] found that 68.4% of the students who read the daily paper would recycle it if there was a central place on campus to do so; 96.6% of the student respondents who lived on campus would recycle their daily paper if there was a drop-off bin on their residence hall floor. A more recent study by Kelly and colleagues [14] found that more individuals on campus said they would recycle if it was made more convenient and if they knew what happened to the recycled items after they were collected.

Limited environmental awareness and college campus culture are barriers to campus greening [15]. A survey conducted at Michigan State University found that university members believed recycling was important but they had limited knowledge regarding what they could recycle and places on campus where they could recycle [16]. This paper contributes to the current literature on recycling education and health communications campaign development, implementation, and evaluation. The goal of this South Carolina campaign was to increase people’s awareness and knowledge about recycling and about the link between a healthy environment and the public’s health.

## 2. Methods

Nine graduate level applied health communications students and their faculty mentor (DBF) at the University of South Carolina secured a National Public Health Week grant to carry out the “This is Public Health” campaign funded by the Association of Schools of Public Health. Campaign activities were introduced to the University of South Carolina Columbia campus and the Greater Columbia, SC area during the April 2008 National Public Health Week. The study involved a pre/post-test evaluation of a university and community outreach program about recycling and healthy environments.

### 2.1. Research Sites and Participants

Since the University of South Carolina is a central part of the community, the campaign targeted both the university community and surrounding area. Campaign events took place at the South Carolina Public Health Association’s (SCPHA) annual Public Health Month kick-off event at the state museum, the university student union, and a local public library. There is a considerable amount of literature supporting the growing role of the library in providing consumers with public health information [17–19].

### 2.2. Campaign Week Activities and Media Promotion

The University of South Carolina “This is Public Health” campaign team partnered with the SCPHA to participate in their annual Public Health Month Kick Off event which was held at the state museum. The campaign team held an information table where attendees were asked to complete the campaign survey. Each survey participant received a recycling bin and educational information about recycling. This event provided an excellent introduction to the campaign and an opportunity to integrate campaign activities into the community with a public health partner.

The local library is an active supporter of recycling, engaging in recycling activities on a daily basis, and promotional events such as recycling old phonebooks at the beginning of each year. As part of the campaign activities, the team set up information tables in the library’s activity room. In exchange for completing the campaign survey, participants received a reusable tote bag with educational information about recycling. The library also helped to promote the recycling campaign by advertising the campaign on the library’s webpage, in a local newspaper of community events, and in a school newsletter. A local news affiliate aired campaign activities at the library during National Public Health Week.

The university events were held at the student union building, a busy location frequently visited by on-campus and commuter students, staff, and faculty. Campaign activities took place during the busy meal hours. A campaign booth was set up and run by two to three members of the campaign team. Similar to the events at the library, individuals completing a survey received a reusable grocery bag that contained educational information about recycling. Campaign events taking place at the student union were promoted in the university student newspaper and on the campus radio station. One member of the campaign team also dressed as “Can Guy”, a member of the SC DHEC’s “Recycling Guys”.

Using the “This is Public Health” logo, the campaign team developed several promotional products. Two banners promoting the “This is Public Health: Recycling Counts!” message were created and displayed at all events and in the university’s public health building. Recyclable grocery bags with the logo were designed and distributed along with educational information about recycling. T-shirts were designed for the campaign team members to wear during the events so they were easily identifiable. The SCPHA and SC DHEC provided the campaign team with a multitude of educational resources. A brochure describing the importance of recycling and the link between health and recycling, a Public Health Week question and answer fact sheet, and county resource lists with recycling locations and materials accepted for recycling at various recycling depots were also distributed during the campaign week. Finally, participants were given bookmarks; mouse pads; pencils made of recycled jeans, money and paper; and coloring and activity books about recycling.

University-wide and local media was used to promote the campaign events. Two articles were posted on the university’s website, a letter to the editor was published in The State newspaper, and advertisements were run on the university’s daybook of events, cable network and telephone hold message. The campaign faculty mentor and one student also promoted the campaign in a radio interview on WGCV AM’s “Health, Wealth, and Happiness” program.

### 2.3. Data Collection: Self-Administered Surveys

A recycling survey consisting of ten Likert-type, multiple-choice, and fill-in-the-blank questions was developed to assess participants’ recycling behaviors, and information sources, knowledge, and attitudes about recycling and the environment. Pencil-paper surveys were administered during National Public Health Week at all campaign related activities, booths, and events (described in section 2.4). Survey questions were adapted from a SC Department of Health and Environmental Control (SC DHEC) Office of Solid Waste Reduction & Recycling postcard survey and the Association of Schools of Public Health’s National Public Health Week sample list of questions e-mailed to “This is Public Health” grantees. A few additional questions were developed pertaining to recycling attitudes, knowledge, and behaviors. Survey questions were pilot tested with university students for clarity, word choice, and survey length.

Follow-up surveys were sent to participants three weeks after National Public Health Week campaign activities to determine if people’s awareness about recycling and recycling behaviors had changed as a result of the campaign. Participants’ pre/post surveys were matched using a numeric code. Once all completed post-surveys were received, participants were entered into a raffle. Individuals from the museum event were entered to win one of 10 reusable grocery bags, and participants from the student union and library were entered to win one of 15 gift cards.

### 2.4. Data Analysis

Survey results were analyzed in SPSS 16.0. Descriptive statistics (*i.e.*, frequencies) were generated. Chi-squares were used to examine survey results according to data collection site (university vs. community). Paired t-tests were used to determine differences in pre- and post-survey results for continuous variables (e.g., frequency of recycling pre-campaign vs. post-campaign). Statistical significance was set at *P < 0.05.*

### 2.5. Campaign Framework

**Figure 1.**
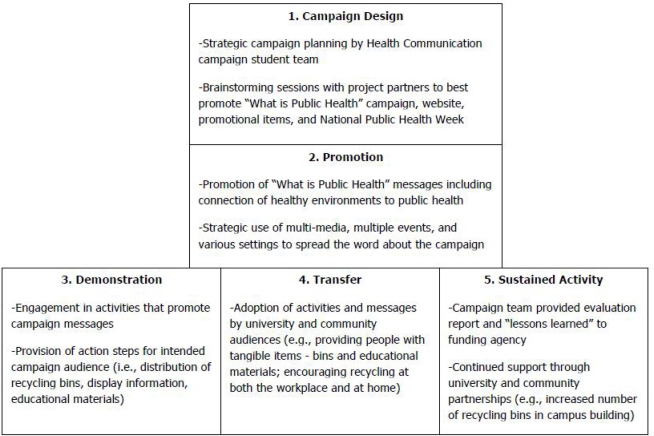
“This is Public Health” Health Communications Campaign Model. * * Adapted from [20].

Development, implementation, and evaluation of the recycling campaign were based on Rudd and colleagues’ five-stage sustainability model (campaign design, promotion, demonstration, transfer of knowledge/skills, and sustainability) as shown in Figure 1 [20]. Principles of The Health Communication Unit’s *Twelve Steps to Developing a Health Communication Campaign* were also considered in campaign development and event promotion and organization [21].

When recycling was identified as the target behavior by the campaign team, “Recycling Counts!” was added to the “This Is Public Health” branding that had already been established by the funding agency. Existing promotional resources about recycling from reputable organizations (SCPHA and SC DHEC) were identified and featured heavily in campaign materials. Demonstration activities included the distribution of recycling bins and educational resources such as pamphlets specifying locations of recycling facilities to the campaign audience. Once the campaign ended, the recycling bins given to the audience and placed in the university setting would be used as a means to sustain recycling behaviors.

## 3. Results

### 3.1. Participation Results

The events and recycling efforts were well received by participants at all three campaign locations (museum, library, student union). To determine recycling habits of individuals at the university and in the Columbia, SC community, a total of 258 individuals were surveyed during campaign week: 147 at the university and 111 in the community. Fifty participants completed surveys at the museum campaign kick-off events, 61 at the library, and 147 at the university student union. The public library reached a diverse audience such as families with home-schooled children, military families, senior citizens, and children congregating for after school programming. Campaign promotion at the student union attracted the most students, faculty, and staff during lunchtime hours (11 am–1 pm).

### 3.2. Campaign Survey Findings

The initial campaign week survey results revealed that 114 of the 147 university participants (77.6%) recycled plastic most often followed by paper (76.2%) and glass (59.2%). Community participants reported recycling aluminum and tin cans most often (84.7%) followed by plastic (78.4%), and paper (73.0%) items (Table 1). The majority of participants from the university (53.7%) “agreed” that they made a conscious effort to recycle; fewer people “strongly agreed” (32.7%) or “disagreed” (8.8%) with this statement. Most community participants (54.1%) “strongly agreed” that they made a conscious effort to recycle; fewer “agreed” (39.6%); very few “disagreed” (3.6%). Few participants at the university (39/147 or 26.5%) and in the community (40/111 or 36.0%) stated that they were “very familiar” with their recycling schedule and guidelines. Overall, 36.4% (n = 94) of all participants were “somewhat familiar” or “unfamiliar” with guidelines.

The most commonly reported barrier to recycling in both the community (9.9%) and on campus (16.3%) was inconvenience regarding location of recycling facilities. A significantly greater percentage of university than community participants (15.6% vs. 7.2%) reported not having a recycling bin / limited access to bins as a key barrier to recycling (p = 0.039). Although selected less often, other barriers included time (University: 3.4%; Community: 1.8%), not knowing the recycling day (University: 5.4%; Community: 1.8%), not knowing which items to recycle (University: 3.4%; Community: 3.6%), and not caring (University: 3.4%; Community: 1.8%).

When asked from which media sources they heard / read about recycling and the environment, most people selected television and newspapers (Table 2). A significantly greater percentage of university participants learned about the benefits of recycling from the Internet (p = 0.025).

Finally, quite a few participants at both the university (70.7%) and in the community (76.6%) “strongly agreed” that the environment has an effect on human health. In addition, 79 (53.7%) university and 75 (67.6%) community members “strongly agreed” that recycling was indeed related to public health.

Follow-up surveys were sent to participants three weeks following National Public Health Week (*i.e.*, campaign implementation week) to determine if their awareness about recycling and recycling behaviors had changed as a result of the campaign. Twenty-six individuals completed the follow-up survey. Paired t-tests showed significant increases in the number of times per month that individuals recycled paper, plastic, glass, and cans (Table 3). The majority of participants (88.4%) “agreed” or “strongly agreed” that they had greater awareness about the link between recycling and public health as a result of the campaign. Furthermore, many participants (61.6%) “agreed” or “strongly agreed” that their recycling efforts increased as a result of the information they received from the “This is Public Health: Recycling Counts!” campaign.

## 4. Discussion and Conclusions

National attention has turned toward environmental sustainability. In 2009, the U.S. Congress passed the American Clean Energy and Security Act with a goal to reduce greenhouse gases by 17% by the year 2020 [22]. Recycling efforts can effectively reduce adverse health effects caused by environmentally hazardous materials. The recycling industry also benefits local revenue, and contributes to employment and business expansion [6]. The purpose of this pilot study was to implement a health communications campaign about recycling during National Public Health Week 2008. Campaign goals were to increase knowledge and awareness about recycling and increase positive attitudes toward recycling, with the hope of increasing recycling behaviors. The impact of the campaign was strengthened through effective engagement with community partners. Survey results identified inconvenience of recycling facility locations as a key barrier to recycling by both university and community participants.

### 4.1. Recycling Knowledge and Behavior

General recycling information and recycling surveys were distributed and collected at predetermined locations on the university campus and within the community at a museum and local library. While participants did engage in some recycling and they understood the importance of recycling, many had not taken the time to familiarize themselves with recycling schedules and guidelines. Thus, the value that participants place on recycling may not be sufficient to promote recycling behaviors. Results from Kaplowitz and colleagues’ research [16] were similar in that recycling was hindered by a lack of knowledge specifically among college students regarding university recycling policies and schedules. Although recycling programs have become more common at universities, there is a need to increase public awareness of recycling policies regarding materials that can be recycled [11].

### 4.2. Sources of Recycling Information

Overall, university and community members reported that they obtained recycling information primarily from television and newspaper sources. The challenge for most health communications programs is to tailor health messages effectively and to use appropriate media sources in order to maximize target reach with minimal program cost [23]. Although television is a popular form of media, the cost to broadcast recycling messages (e.g., public service announcements) may not be practical for grassroots and unfunded projects. This study incorporated several types of free or low cost local and university media outlets (broadcast and print) to increase recycling awareness.

### 4.3. Barriers to Recycling

Overall, survey results revealed that individuals from the university and the community are recycling. Cans, plastic, and paper are the main materials being recycled. However, a key barrier to recycling was that it was not convenient. This result is consistent with previous research [24]. More accessible and convenient recycling locations and greater distribution of free recycling bins will be required to increase recycling rates on campus and in the community [11].

### 4.4. Study Limitations

This study has limitations. First, funding only permitted implementation of a brief campaign, thus it is unlikely that this project had a significant long term impact on the target population’s recycling behaviors. Second, pre-testing campaign messages is important when developing any communication based project. In this campaign, however, the “This is Public Health” slogan was provided to the team by the funding agency. “Recycling Counts!” was added to the initial slogan to more effectively communicate the connection between recycling, the environment, and public health. Third, demographic variables other than campaign site visited (museum, library, or student union) were not collected, thus recycling knowledge and behavior by age, gender, race / ethnicity or role (e.g., staff versus student) could not be examined. Fourth, a very small sample of individuals completed the post-campaign survey (n = 26), and a longer-term, more rigorous evaluation was not conducted as a part of this pilot campaign project. Finally, an assessment specifically of the effectiveness of the media promotion of the campaign was not conducted. It is recommended that an assessment of media messages is conducted for a larger recycling campaign.

### 4.5. Study Strengths and Implications

Despite study limitations, the campaign received attention through university and local media and was strengthened considerably by collaborative partnerships with the university, community, media, and state government. Partnerships are extremely important in campaign development [21]. Another positive outcome from this pilot project was with regard to sustainability. An informal partnership was established between the campaign team and university facilities, resulting in greater recycling opportunities and placement of an increased number of recycling bins in a public health building on the university campus.

The amount of paper Americans throw away each year is equivalent to one billion trees [1]. If all of the newspapers in the U.S. were recycled, 250 million trees would be saved each year. Recycling programs can contribute to improved public health by reducing unnecessary waste which harms the environment and in turn, negatively impacts global health outcomes [25]. Recycling centers must be accessible to its citizens and more recycling bins need to be located in work places and other public areas. Future large-scale media and educational campaigns should inform and promote recycling to facilitate personal, community, and societal changes [26,27]. Findings from this pilot study suggest that a theoretically driven health communications campaign to improve recycling behaviors may be effective in both university and community settings.

## Figures and Tables

**Table 1.** Types of material recycled, results from initial campaign week survey (N = 258).

**Material**	**University (N = 147)**	**Community (N = 111)**

	No. (% of 147 total)	No. (% of 111 total)

Glass	87 (59.2)	54 (48.6)
Plastic	114 (77.6)	87 (78.4)
Paper	112 (76.2)	81 (73.0)
Cardboard	61 (41.5)	51 (45.9)
Batteries	32 (21.8)	30 (27.0)
Cans	111 (75.5)	94 (84.7)
Compost	17 (11.6)	26 (23.4)

**Table 2.** Media sources consulted for recycling information, results from initial campaign week survey (N = 258).

**Recycling information source**	**University (N = 147)**	**Community (N = 111)**

	No. (% of 147 total)	No. (% of 111 total)

Television	120 (81.6)	91 (82.0)
Newspaper	90 (61.2)	59 (53.2)
Radio	54 (36.7)	47 (42.3)
Internet	83 (56.5)	47 (42.3)
Magazine	55 (37.4)	43 (38.7)
No information received	9 (6.1)	13 (11.7)

**Table 3.** Average number of times participants recycled per month: a pre/post campaign comparison (N = 26).

**Item**	**Mean (SD)**		**Significance Level**

	***Pre-Campaign***	***Post-Campaign***	***P-values***

**Paper**	8.46 (10.19)	12.00 (15.78)	P = 0.026
**Plastic**	7.13 (8.66)	11.50 (13.26)	P = 0.018
**Glass**	5.17 (7.01)	5.83 (9.51)	P = 0.032
**Cans**	6.96 (8.76)	9.96 (11.94)	P = 0.023
